# Silk Hydrogel Substrate Stress Relaxation Primes Mesenchymal Stem Cell Behavior in 2D

**DOI:** 10.1021/acsami.1c09071

**Published:** 2021-06-25

**Authors:** Suttinee Phuagkhaopong, Luís Mendes, Katrin Müller, Manja Wobus, Martin Bornhäuser, Hilary V. O. Carswell, Iola F. Duarte, F. Philipp Seib

**Affiliations:** † Strathclyde Institute of Pharmacy and Biomedical Sciences, University of Strathclyde, Glasgow G4 0RE, U.K.; ‡ CICECO − Aveiro Institute of Materials, Department of Chemistry, University of Aveiro, Aveiro 3810-193, Portugal; § University Hospital Carl Gustav Carus, Technical University Dresden, Dresden 01307, Germany; ∥ Center for Regenerative Therapies Dresden (CRTD), Technical University Dresden, Dresden 01307, Germany; ⊥ EPSRC Future Manufacturing Research Hub for Continuous Manufacturing and Advanced Crystallisation (CMAC), University of Strathclyde, Technology and Innovation Centre, Glasgow G1 1RD, U.K.; ▲ Leibniz Institute of Polymer Research Dresden, Max Bergmann Center of Biomaterials Dresden, Dresden 01069, Germany

**Keywords:** silk fibroin, B. mori, viscoelastic, substrate mechanics, mesenchymal stromal cells

## Abstract

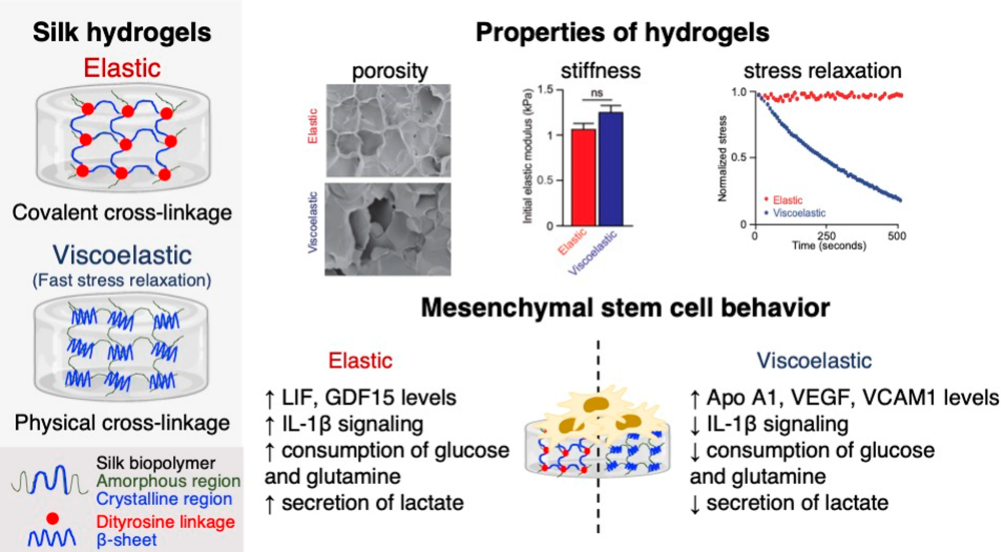

Tissue-mimetic silk hydrogels are being explored for diverse healthcare applications, including stem cell delivery. However, the impact of stress relaxation of silk hydrogels on human mesenchymal stem cell (MSC) biology is poorly defined. The aim of this study was to fabricate silk hydrogels with tuned mechanical properties that allowed the regulation of MSC biology in two dimensions. The silk content and stiffness of both elastic and viscoelastic silk hydrogels were kept constant to permit direct comparisons. Gene expression of *IL-1β*, *IL-6*, *LIF*, *BMP-6*, *BMP-7*, and *protein tyrosine phosphatase receptor type C* were substantially higher in MSCs cultured on elastic hydrogels than those on viscoelastic hydrogels, whereas this pattern was reversed for *insulin*, *HNF-1A*, and *SOX-2*. Protein expression was also mechanosensitive and the elastic cultures showed strong activation of *IL-1β* signaling in response to hydrogel mechanics. An elastic substrate also induced higher consumption of glucose and aspartate, coupled with a higher secretion of lactate, than was observed in MSCs grown on viscoelastic substrate. However, both silk hydrogels changed the magnitude of consumption of glucose, pyruvate, glutamine, and aspartate, and also metabolite secretion, resulting in an overall lower metabolic activity than that found in control cells. Together, these findings describe how stress relaxation impacts the overall biology of MSCs cultured on silk hydrogels.

## 1 Introduction

Human mesenchymal stem cells (MSCs) are widely explored in the clinic because they can differentiate into mesenchymal linages of bone, cartilage, or fat for the repair of injured tissues.^1^ However, MSCs also produce a myriad of paracrine and trophic factors that increase their therapeutic versatility as potent immunosuppressive and anti-inflammatory mediators. One example is the clinical use of MSCs to treat Graft-versus-Host disease.^2^ However, MSCs delivered by simple intravenous injection rapidly undergo perforin-dependent apoptosis within cytotoxic recipient tissues.^3^ Therefore, preclinical studies are now exploring delivery strategies that include bioengineered scaffolds in an effort to improve MSC function. For example, encapsulating MSCs into alginate hydrogels prior to intravenous dosing in mice can change the pharmacokinetic parameters and increase the elimination half-life, extend MSC survival, and improve allogenic bone marrow engraftment.^4^

MSCs are responsive to many different cues, including physical features such as niche geometry and mechanics.^5^ For example, substrate stiffness is a key regulator of MSC linage commitment both in two^6^ and three dimensions.^7^ However, the wider mechanical space also impacts cell behavior,^8^ including MSC biology.^7^ Therefore, the stiffness and the substrate stress relaxation are key physical parameters that dictate MSC biology. Both fundamental and applied studies have used a range of materials, including Matrigel, alginate, polyethylene glycol, and polyacrylamide-based hydrogels, to tune the mechanics of transplanted MSCs. However, many of these materials are not suited for clinical applications.

The use of materials with potential for clinical translation is particularly timely. One promising contender for MSC application is silk.^9^ The silk fiber is clinically approved for use in humans and has a robust track record.^10^*Bombyx mori* silk fibers have been used for millennia as a suture material that is renowned for its availability, mechanical robustness, knot strength, ease of handling, and biocompatibility. However, silk has wider potential extending far beyond sutures. Over the past 2 decades, interest has been renewed in biomedical applications of silk, both in its spun fiber format and in a fully reverse-engineered liquid format.^10^ For example, knitted silk surgical meshes (SERI Surgical Scaffold, Sofregen Inc., MA, USA) fabricated from naturally spun silk fibers or from silk hydrogels (e.g., a bulking agent for vocal fold insufficiency, Silk Voice, Sofregen Inc.) derived from liquid silk have recently been approved for use in humans.^10,11^ Silk fibers, films, scaffolds, and hydrogels have been combined with MSCs to explore potential tissue engineering applications in two, three, and four dimensions.^9^ This includes the use of silk-based materials for use as scaffolds to deploy tumor-homing MSCs armed with genetically introduced therapeutic genes.^12^ Emerging evidence currently supports the use of silk hydrogels for MSC applications.^13^ Therefore, controlling silk hydrogel function is now a key research area.

Silk hydrogels can be readily tuned by tailoring the silk protein concentration and composition, in addition to manipulating the cross-linking mechanism and density.^14−17^ The resulting silk hydrogels have been explored in a broad spectrum of tissues and applications (e.g., skin,^18^ bone,^19^ cartilage,^20^ muscle,^21^ pancreas,^22^ and brain^23,24^). For example, embedding MSCs within a physically cross-linked 4% (w/v) silk hydrogel provided the best cell proliferation, whereas a higher silk content (8 and 12%, w/v) impaired cell proliferation.^14^

The ability to trigger the solution–gel transition was explored as a benefit for stereotactic injections.^24,25^ MSCs retained their viability in the solution phase, allowing minimally invasive administration deep into the target site, where the administered dose completed the solution-gel transition in situ. Seminal work by González-Nieto and co-workers^23^ subsequently demonstrated the suitability of this delivery technology for the administration of MSCs into the epicenter of a cortical stroke region in mouse brains. Mice treated with MSCs showed reduced cortical tissue loss, improved MSC survival, and cortical rewiring with partial functional recovery. In vitro studies have shown that MSCs exposed to silk hydrogels respond by downregulation stromal cell-derived factor 1 (SDF-1), brain-derived neurotrophic factor (BDNF), and vascular endothelial growth factor (VEGF), while increasing transforming growth factor beta-1 (TGF-β1) secretion.^26^

Despite the increasing popularity of silk for tissue engineering applications,^10^ the proposed use of silk hydrogels as carriers for cell therapies (i.e., advanced therapy medicinal products)^13^ and the fundamental understanding of silk hydrogel performance^27^ and its impact on MSC biology remain limited. This also includes the performance of MSCs in two dimensions. In particular, the impact of silk hydrogel stress relaxation on human MSC gene expression and the composition of the secretome and metabolome is unknown. Therefore, the aim of the present study was to exploit chemical and physical cross-linking to fabricate elastic and viscoelastic silk hydrogels, respectively, that had equivalent silk content and stiffness. These hydrogels were used to form two-dimensional cell culture substrates. The cell choice for this study was primary human MSCs obtained from four different healthy donors to minimize source variability.

## 2 Experimental Section

### 2.1 Silk Hydrogel Manufacture

The silk fibroin solution was prepared from *Bombyx mori* cocoons, as detailed elsewhere.^28^ Briefly, dried cocoons were cut into 5 × 5 mm pieces and 5 g samples were degummed with 2 L of 25 mM Na_2_CO_3_ for 60 min (degumming time). The degummed fibers were then rinsed with 1 L of Milli-Q water for 20 min; this process was repeated twice more. The extracted silk fibroin was air-dried in a fume hood overnight, then dissolved in 9.3 M LiBr [silk/LiBr ratio of 1:4 (g/mL)], and heated in an oven at 60 °C for up to 4 h. The dissolved silk fibroin was then transferred to a dialysis cassette (molecular weight cutoff 3500 Da; Thermo Fisher Scientific Inc., Waltham, MA, USA) and dialyzed against Milli-Q water, with six water changes over 48 h. The dialyzed silk fibroin solution was then collected, centrifuged twice at 9500*g* for 20 min to remove any aggregates, and stored at 4 °C until use. The silk fibroin concentration was calculated gravimetrically.

Silk hydrogels denoted as viscoelastic were manufactured by sonication using a digitally controlled probe sonicator (Sonoplus HD 2070, Bandelin, Berlin, Germany) fitted with a 23 cm long sonication tip (0.3 cm diameter tip and tapered over 8 cm). A total volume of 4 mL of 4% (w/v) silk fibroin solution was added to 15 mL Falcon tubes (1.4 cm diameter and 11 cm long) (Greiner Bio-One GmbH, Kremsmünster, Austria) and exposed to a 30% amplitude for two or three sonication cycles on ice (one cycle consisted of 30 s on and 30 s off) to induce the solution–gel transition.

Silk hydrogels denoted as elastic were generated by dityrosine cross-linking^29^ using horseradish peroxidase (HRP) (Sigma-Aldrich). Unless stated otherwise, chemical cross-linking was performed with 2.25 U of freshly prepared HRP for every 1 mg of silk fibroin protein. Briefly, 1 mL of 4% (w/v) silk fibroin solution was added to a 1.5 mL Eppendorf tube (Fisher Scientific), followed by 150 μL of HRP (600 U/mL) and 150 μL of fresh 0.3% H_2_O_2_, followed by gentle mixing. The mixtures were then stored at 37 °C until gels were formed.

### 2.2 Dityrosine Bond Monitoring

Elastic silk hydrogel samples were prepared as described above. The tyrosine fluorescence signal was monitored using an excitation wavelength of 310 ± 20 nm and emission wavelength of 410 ± 20 nm (FP-6500 spectrofluorometer, Jasco International CO., Ltd., Japan). Dityrosine fluorescence emission was monitored between 350 and 550 nm. The scan rate was set at 200 nm/min, with 2 nm data intervals. The photomultiplier tube detector voltage was fixed. A silk solution without HRP and H_2_O_2_ was used as a control.

### 2.3 Mechanical Properties Analysis by Rheology

Samples were prepared in silicone molds forming a 20 mm diameter with an average thickness of 4 mm. The hydrogels were then subjected to rheological characterization at 25 °C (HAAKE MARS rheometer, Thermo Fisher Scientific, UK) using stainless steel parallel plates with a 20 mm diameter and appropriate gap size. The storage modulus (*G*′) was recorded continuously using a time sweep over a strain of 0.01–100% at a frequency of 1.0 Hz. Subsequently, the rate of stress relaxation (loss modulus *G*′′) was determined at a fixed gap width and a 15% strain to mimic the human extracellular matrix.^7^ The resulting stress was monitored every 10 s for a total of 500 s. Stress was normalized by the initial stress, and the half stress relaxation time (τ_1/2_), which is the time corresponding to half of the initial stress, was then calculated (as detailed previously^7^). Before measurement, all samples were equilibriated for 20 min and shielded to minimize water evaporation and drying.

### 2.4 Secondary Conformation Analysis by Fourier Transform Infrared Spectroscopy (FTIR)

Samples were frozen overnight at −20 °C and lyophilized (Epsilon 2-4 LSCplus, Christ, Germany). The secondary structure of the dried samples was assessed using a TENSOR II FTIR spectrometer (Bruker Optik GmbH, Ettlingen, Germany) with 128 scans at a 4 cm^–1^ resolution over the wavenumber range of 400–4000 cm^–1^, and the secondary structures were assigned as detailed elsewhere.^30^ Briefly, the amide I region (1595–1705 cm^–1^) was identified and deconvoluted: 1605–1615 cm^–1^ as side chains, 1616–1637 and 1697–1703 cm^–1^ as β-sheet structures, 1638–1655 cm^–1^ as random coil structures, 1656–1662 cm^–1^ as α-helical bands, and 1663–1696 cm^–1^ as β-turns. All spectra were normalized and corrected for water signals. OriginPro 9.0 software was used to peak fit the amide I region of all spectra. The peak full-width at half-maximum was maintained at a fixed value to avoid overfitting the data. Air-dried and 70% ethanol-treated silk films were used as reference samples for low and high β-sheet content, respectively.

### 2.5 Scanning Electron Microscopy

Silk hydrogels were attached to an electrically conducting sticky carbon patch (Agar Scientific, UK), mounted on aluminum stubs, and freeze-dried overnight. Samples were sputter-coated with 15 nm of gold using an ACE200 low-vacuum sputter coater (Leica Microsystems, Wetzlar, Germany). The morphology of the silk hydrogels was imaged by scanning electron microscopy (SEM) using a FE-SEM SU6600 instrument (Hitachi High Technologies, Krefeld, Germany) with a 5 kV accelerating voltage.

### 2.6 Immunodetection of Adsorbed Protein

Human fibronectin (FN) (Sigma-Aldrich, St. Louis, USA) was reconstituted to a final concentration of 10 and 100 ng/mL in PBS (pH 7.4). Prior to protein adsorption, the hydrogel surfaces were rinsed twice with PBS for 10 min. The prepared FN solutions (400 μL) were applied to the hydrogel surface, and the samples were sealed to minimize evaporation and were incubated at 37 °C for 1, 3, 6, and 24 h. A 250 kDa fluorescein isothiocyanate-labeled dextran (2 mg/mL, Sigma-Aldrich) was used as a reference control to account for possible differences between the hydrogels. After the indicated incubation time, the remaining solution was collected and centrifuged at 5000*g* for 5 min. The surface density of adsorbed fibronectin was calculated by measuring the depletion of FN from the solution using a human FN ELISA kit (R&D Systems, USA), according to the manufacturer’s protocol. The data were normalized using the dextran control. The concentration of dextran was determined using a fluorescence plate reader (excitation, 485 nm; emission, 528 nm).

### 2.7 Cell Culture and Viability

Human primary MSCs were isolated from bone marrow aspirates of healthy donors after we obtained their informed consent. The Institutional Review Board of the Medical Faculty at the University Hospital Dresden approved the study. Human MSCs were expanded and characterized, as detailed previously.^31^ For all silk hydrogel studies, MSCs were used for up to three passages. Elastic and viscoelastic silk hydrogels were prepared as described above but using filter-sterilized stocks (prepared with a 33 mm Millex-GP syringe filter fitted with a poly(ether sulfone) membrane with 0.22 μm pores). A 50 μL sample undergoing the solution–gel transition was pipetted into each well of a tissue culture-treated polystyrene 96-well plate (well surface area 0.32 cm^2^) (Corning Inc., New York, USA). Plates were transferred to a cell incubator (humidified atmosphere of 5% CO_2_ at 37 °C) for 3 h to allow completion of the silk solution–gel transition. A 200 μL volume of low glucose Dulbecco’s modified Eagle medium (DMEM) (Thermo Fisher Scientific) supplemented with 10% v/v fetal bovine serum (Stem Cell Technologies, France), 1% GlutaMAX (Thermo Fisher Scientific), 50 U/mL penicillin, and 50 μg/mL streptomycin (Thermo Fisher Scientific) was carefully added onto the top of the silk hydrogels. The medium was changed twice and the silk hydrogels were allowed to equilibrate overnight in the cell incubator. The next day, the medium was removed and human MSCs in complete DMEM were seeded on top of the hydrogels at a density of 5000 cells/cm^2^ in 200 μL of medium (i.e., two-dimensional culture). The medium was changed at days 3 and 7.

Cell viability was measured at days 1–14. In brief, the medium was removed and replaced with fresh DMEM medium, and 25 μL of resazurin was added (440 μM stock in PBS, Thermo Fisher Scientific). The cells were allowed to metabolize the substrate for 4 h, and then 100 μL of the supernatant was transferred into a black 96-well plate (Sigma-Aldrich). The fluorescence was measured with a fluorescence plate reader (POLARstar Omega BMG LABTECH GmbH, Ortenburg, Germany) by fixing the photo multiplier tube and setting the excitation and emission filters at 560 and 590 nm, respectively. Blank hydrogels from the same time points were used as controls to subtract background fluorescence.

### 2.8 Cell Proliferation

DNA concentration was measured at days 1–14 using the Quant-iTTM PicoGreen kit (Invitrogen-Life Technologies, Grand Island, NY, USA). In brief, culture medium was removed and replaced with 200 μL of PBS for 3 h. The samples were homogenized and digested with 200 μL of papain buffer solution (5 mg/mL papain, 2 mM cysteine, 50 mM sodium phosphate, and 2 mM ethylenediaminetetraacetic acid, pH 6.5, in nuclease-free water) at 60 °C for 16 h. The papain-digested samples were collected and centrifuged for 5 min at 13 000*g* to eliminate cellular debris. The supernatants were collected and dDNA was quantified with the Quant-iTTM PicoGreen kit, following the manufacturer’s protocol. Blank hydrogels from the same time points were used as controls to account for background fluorescence.

### 2.9 Cell Staining

hMSCs were cultured on silk hydrogel substrates in four-chamber slides for 3 days. Cell-seeded hydrogels were fixed in 4% v/v methanol-free formaldehyde, permeabilized in 0.1% v/v Triton-X 100 for 15 min, and blocked in 1% w/v bovine serum albumin (BSA; Sigma-Aldrich) for 1 h at room temperature. Hydrogels were then incubated overnight at 4 °C with primary antibodies against YAP (rabbit polyclonal anti-YAP1 antibody, 1:50 dilution in PBS/BSA 1% w/v, Abcam, UK). The hydrogels were given three 10 min washes with PBS and then incubated in the dark at room temperature for 2 h with secondary antibodies (AlexaFluor 555 goat antirabbit IgG, 1:500 dilution in PBS/BSA 1% w/v, Abcam). The hydrogels were then rinsed three times with PBS and stained with a Hoechst 33342 nuclear stain (1:1000 dilution in PBS, Cayman Chemicals, USA) for 10 min, followed by two rinses with PBS. For actin filament visualization, cells were stained with a phalloidin-Alexa488 dye, according to the manufacturer’s instructions (Thermo Fisher Scientific Inc., Waltham, MA, USA). In brief, cells were washed and fixed as detailed above. Next, cells were incubated at room temperature with phalloidin-Alexa 488 at a final concentration of 0.165 μM in PBS for 1 h to stain the β-actin cytoskeleton. Stained hydrogels were stored in the dark at 4 °C until imaging (Epifluorescence upright microscope, Nikon Eclipse E600). Exposure time and other image settings for each respective channel were held constant during imaging. Images were adjusted, processed, and analyzed in ImageJ 1.51s (National Institutes of Health, Bethesda, USA).

### 2.10 Image Analyses

For investigation of the cytoskeletal organization of the cells, single cells were manually traced from fluorescent actin images. Area, perimeter, fit ellipse, and shape descriptors were quantified by ImageJ. Shape descriptor values were then used to calculate four metrics as detailed elsewhere:^32^ namely, cell area (π × radius^2^), circularity ((4 × π × area)/(perimeter^2^)), roundness ((4 × area)/(π × major axis length^2^)), and aspect ratio (major axis length/minor axis length) (Figure S1, Supporting Information). With these metrics, a line and a circle have values of 0 and 1, respectively. For YAP/TAZ staining, the nuclear to cytoplasmic ratio was calculated with the formula: nuclear YAP = (nuclear YAP intensity/area of nucleus)/(cytosolic YAP intensity/area of cytosol) (detailed previously^33^).

### 2.11 Quantitative Real-Time Polymerase Chain Reaction (qRT-PCR)

Gene expression of hMSCs cultured on different elastic hydrogels was assessed using a similar cell culture approach as detailed above. Briefly, the medium was removed, followed by washing with PBS twice, and then cells were harvested with Accutase (Thermo Fisher Scientific). Total RNA was extracted from hMSCs using MicroRNeasy Kit (Qiagen), according to the manufacturer’s instructions. Quantification of RNA was performed on a Nanodrop 2000 spectrophotometer (Thermo Scientific). RNA samples from each donor were used to determine the expression of nine selected target genes. The cDNA was synthesized from 1 μg of total RNA using the QuantiTect reverse transcription kit (Qiagen, USA). Quantitative RT-PCR was performed using the QuantiTect SYBR Green PCR kit (Qiagen) on an Applied Biosystems 7500 Real-time PCR system (ABI 7500, Applied Biosystems). The targets, which included human *IL-1B*, *IL-6*, *ITGB1*, *ITGV*, *LIF*, *MMP2*, *RhoA*, *VEGFA*, and *VCAM1*, were determined according to the Qiagen guidelines. *GAPDH* and *RPL30* were used as reference genes. The threshold cycle (*C*_t_) value of each target gene was normalized to the expression of two different housekeeping genes (*GAPDH* and *actin-beta*) for the RT^2^Profiler PCR Array and *GAPDH* and *RPL30* for single target primer assays. The difference between the *C*_t_ value of a target gene and the housekeeping genes of cells cultured on hydrogels was subtracted from the difference between the *C*_t_ value of the target gene and housekeeping genes of cells cultured on plasma-treated tissue culture plastic, and it was then expressed as a relative fold change (RFC) according to the 2^–ΔΔ*C*_t_^ method. The relative fold change value of plasma-treated tissue culture plastic was defined as 1. For the RT^2^Profiler PCR Array experiments, extracted RNA from MSCs of the four healthy donors were pooled. Single-stranded complementary DNA (cDNA) was synthesized from 500 ng of total RNA and amplified with RT^2^ PreAMP cDNA synthesis with RT^2^ PreAMP cDNA Synthesis Primer Mix for human mesenchymal stem cells. The RT^2^Profiler PCR Array (PAHS-082Z, Qiagen) was used for transcriptome profiler expression analysis according to the manufacturer’s instructions.

### 2.12 Gene Network and Pathway Analysis

A short list of differentially expressed genes was generated based on the following criteria: mRNAs with *p*-value <0.05 and log2 fold change >2. The transcriptome data set in response to MSC culture was visualized by comparing the MSCs on silk hydrogels to those on a tissue culture plastic. A normalized *z*-score was calculated and a heat map was generated using the R package software (R Studio 3.5.2 version; The R Foundation, Boston, MA; available at r-project.org). Similarly, the short-listed differentially expressed genes were used to perform a core analysis with the IPA software (Qiagen) to identify the upstream regulators of the differentially expressed genes and related canonical pathways that were altered by the culture on silk hydrogels. The IPA core analyses were based on previous knowledge of the associations of upstream regulators and their downstream target genes archived in the Ingenuity Knowledge Base. When mapped to canonical pathways, the pathway that had the highest IPA score was considered the most differentiated from the others. The *p*-values were calculated by Fisher’s exact test for the upstream regulator analysis.

### 2.13 Proteome Profiler Analysis

Protein expression patterns were determined from pooled conditioned culture medium from the four MSC donors. The conditioned medium was collected and centrifuged at 5000*g* for 5 min to remove any cells or cell debris. The supernatant was transferred to new tubes and stored at −80 °C until use. Human cytokine proteome profiler (Panel A, R&D Systems, Minneapolis, MN, USA) was used, according to the manufacturer’s instructions. For the analysis of arrays, blots were threshold-adjusted and analyzed using ImageJ. The intensity for a specific cytokine was then computed by averaging over duplicated spots.

### 2.14 Sample Preparation for Metabolomics and NMR Spectroscopy

The culture medium was collected, clarified by centrifugation at 5000*g* for 5 min, lyophilized, and stored at −80 °C until use. NMR analyses were conducted after reconstituting the freeze-dried medium in 600 μL of deuterated phosphate buffer (100 mM, pH 7.4) containing 0.1 mM 3-(trimethylsilyl)propionate sodium salt, (TSP)-*d*_4_. A 550 μL volume of each sample was transferred into 5 mm NMR tubes, and NMR spectra were acquired on a Bruker Avance III HD 500 spectrometer (University of Aveiro, Portuguese NMR Network) operating at 298 K and 500.13 MHz for ^1^H observation. Standard 1D ^1^H spectra with water presaturation (pulse program “noesypr1d” in the Bruker library) were recorded with a 7002.801 Hz spectral width, 32768 data points, a 2 s relaxation delay, and 512 scans. The spectral processing comprised cosine multiplication (ssb 2), zero-filling to 65536 data points, manual phasing, baseline correction, and chemical shift calibration to the TSP-*d*_4_ signal at δ 0 ppm. Each spectrum was multiplied by a correction factor to account for the different volumes of medium lyophilized and the different cell numbers, which were associated with DNA content.

Metabolites were identified by matching our spectral data to reference spectra in the reference libraries in the Human Metabolome Data Base (HMDB), BBIOREFCODE-2-0-0 (Bruker Biospin, Rheinstetten, Germany) and Chenomx (Edmonton, AB, Canada). Quantitative variations were assessed through spectral integration of selected signals using Amix-Viewer 3.9.15 (Bruker Biospin, Rheinstetten, Germany). For each metabolite, fold changes were calculated relative to respective acellular medium controls. Metabolite variations with an absolute fold change ≥1.05 were classified as consumed or secreted and plotted as a heat map.

### 2.15 Data and Statistical Analyses

Data were analyzed using GraphPad Prism 8.0^@^ (GraphPad Software, La Jolla, CA). Student’s *t* tests were used to analyze sample pairs. One-way analysis of variance (ANOVA) between controls and elastic and viscoelastic silk hydrogels were conducted, followed by Tukey’s multiple comparison post hoc test for multiple samples. Statistical significance was indicated by asterisks in each figure legend and assigned as follows: *p ≤ 0.05 and **p ≤ 0.01. All data were plotted as mean ± standard deviation (SD) and, unless otherwise stated, refer to a minimum of three independent biological repeats.

## 3 Results

### 3.1 Fabrication and Characterization of Silk Hydrogels with Tuned Stress Relaxation

**Figure 1:**
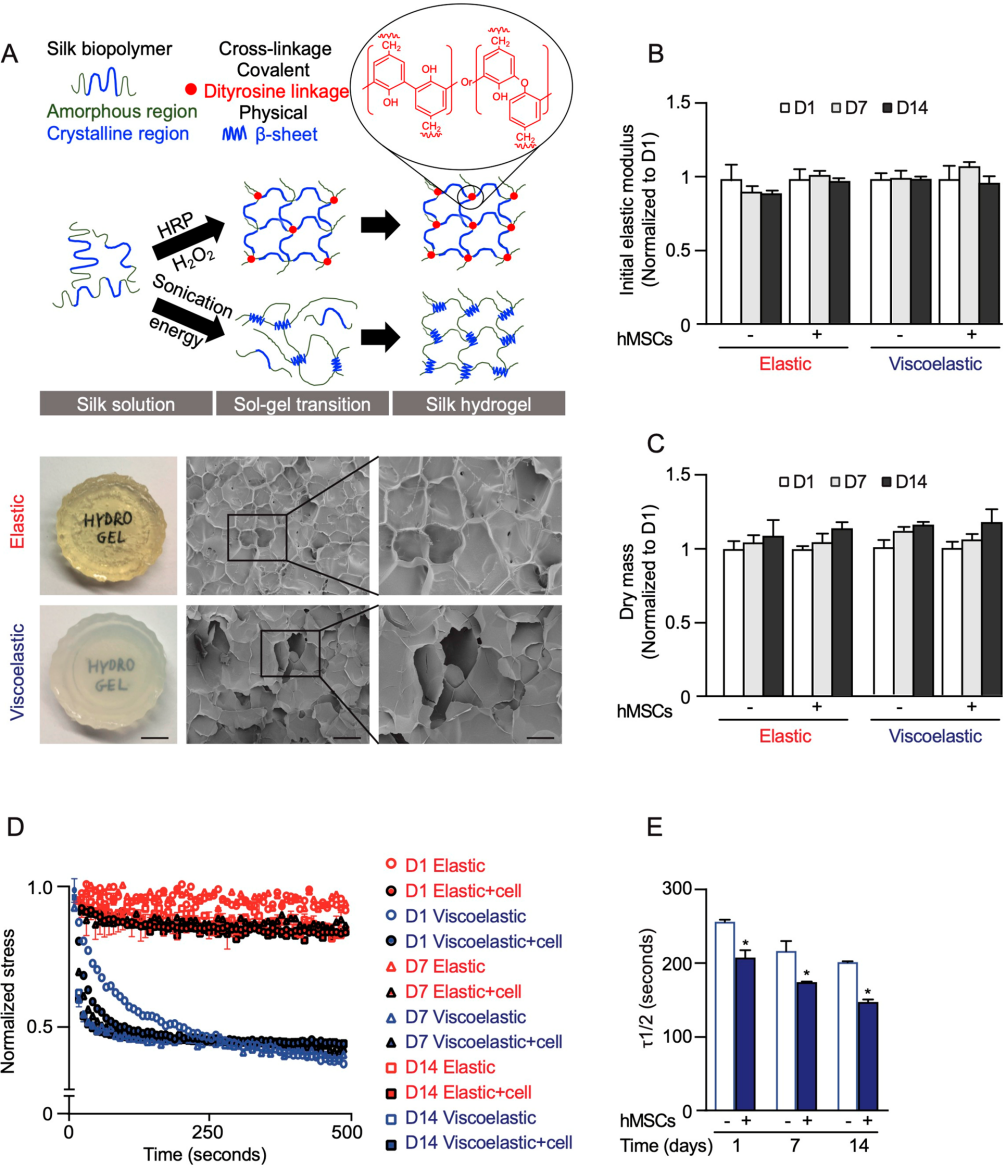
Silk hydrogels used for cell culture studies. (A) Schematic depiction of elastic and viscoelastic silk hydrogel preparation and corresponding macroscopic (scale bar 0.5 cm) and scanning electron microscopic images (scale bar, 200 μm; zoom, 100 μm) of silk hydrogels. (B) Initial elastic modulus (∼1 kPa) and (C) dry mass after 7 and 14 days in the presence and absence of cells, normalized to the value of day 1. (D, E) Stress relaxation under a compressional strain of 15% of the hydrogels in the presence and absence of cells for up to 14 days. Data are presented as mean ± SD, *n* = 5 independent experiments. Error bars are hidden in the plot symbols when not visible. For *p ≤ 0.05, a comparison of silk hydrogel with and without cell culture at the respective time point.

Elastic silk hydrogels were formed using enzymatic cross-linking to yield dityrosine or isodityrosine linkages within the amorphous regions of the silk fibroin heavy chain (Figure 1A). The solution–gel transition was confirmed to be due to covalent cross-linking by analyzing the dityrosine and isodityrosine emission peak spectra by fluorescence spectrophotometry. Dityrosine cross-links were confirmed by a spectral shift from 310 to 410 nm. Linker concentration directly correlated with increased fluorescence intensity and thus cross-link density and ultimately mechanics (Figure S2A). Viscoelastic silk hydrogels were manufactured using sonication energy to induce physical cross-linking, coordinated by hydrogen bond formation between the crystalline regions of the silk fibroin heavy chain (Figure 1A). This change in secondary structure resulted in a conformational transition from a random coil to an antiparallel β-sheet structure (detailed below). Macroscopic examination of both silk hydrogel types indicated that both could be easily manipulated for cell culture while retaining their overall integrity. Elastic silk hydrogels were transparent to visible light, whereas viscoelastic silk hydrogels were opaque because of the abundant β-sheets (detailed below) that caused light scattering. The hydrogels were qualitatively assessed by SEM. Both hydrogel types showed a smooth surface and a similar pore structure (Figure 1A).

The secondary structure of the silk hydrogels was characterized by FTIR. The amide I region (1595–1705 cm^–1^) was identified and deconvoluted. Hydrogel samples were compared to untreated and 70% (v/v) ethanol-treated silk films that served as controls for low (38.78 ± 0.64%) and high (49.27 ± 0.24%) crystallinity, respectively. Spectra of viscoelastic silk hydrogels showed a high β-sheet content (47.05 ± 0.14%) and significantly fewer α-helix and random coil structures when compared to those of the untreated, water-soluble, amorphous silk films. By contrast, the elastic hydrogels had a comparatively small amount of β-sheet structures (21.90%) and side chains (6.44%), but higher percentages of α-helix (8.08%), random coil (38.12%), and turn (25.43%) structures. Importantly, attempts to change the secondary structure of the elastic hydrogels (i.e., by treatment with 70% [v/v] ethanol) only slightly increased the β-sheet content but substantially enhanced the percentage of random coil structures when compared to untreated elastic hydrogels (Figure S2B).

Hydrogel assembly was tuned to generate hydrogels that had an identical silk fibroin content and an identical initial elastic modulus (*G*′) but a different loss modulus (*G*′′), thereby resulting in hydrogels with more elastic or more viscoelastic behaviors. This fine-tuning included titration of the enzymatic cross-linker concentration (2.25–6.75 unit/mg) to generate elastic hydrogels that had an initial modulus of 1 kPa (Figure S3B). Under higher strain levels, the storage modulus (*G*′) of the viscoelastic hydrogels showed a sharp decline, whereas only a very slight drop was observed with the elastic hydrogels. The loss modulus of elastic hydrogels (*G*′′) was very low compared to that of the viscoelastic hydrogels because of their stable covalently cross-linked network, indicating that the formed hydrogels were predominately elastic (Figure 1D, Figure S3A). Stress relaxation tests were performed to quantify the viscoelastic properties. The physically cross-linked silk fibroin displayed fast stress relaxation (τ_1/2_ = ∼250 s), whereas covalently cross-linked hydrogels exhibited a steady response with no stress relaxation over time, as expected for elasticity performance. Overall, these mechanical characteristics justified the selected nomenclature of the respective silk hydrogel. The impact of time and cells on hydrogel mechanics was also characterized. The changes in the mechanical properties and dry masses of these hydrogels were not significant over a time scale of at least 14 days (Figure 1B,C). In the absence of cells, the elastic moduli for both hydrogel types remained statistically the same over 14 days. In the presence of cells, the elastic hydrogels were always stiffer than the control hydrogels (e.g., 0.98 vs 1.12 kPA at day 14). By contrast, the viscoelastic hydrogels became progressively softer, showing a significant drop from 1.24 to 1.05 kPA at day 14 (Figure S3C). Viscoelastic hydrogels with cells also showed progressively faster stress relaxation in a culture, with a 25% reduction at day 14 compared to that at day 1 (Figure 1D,E).

### 3.2 Response of MSC Spreading and Proliferation by Substrate Mechanics

**Figure 2:**
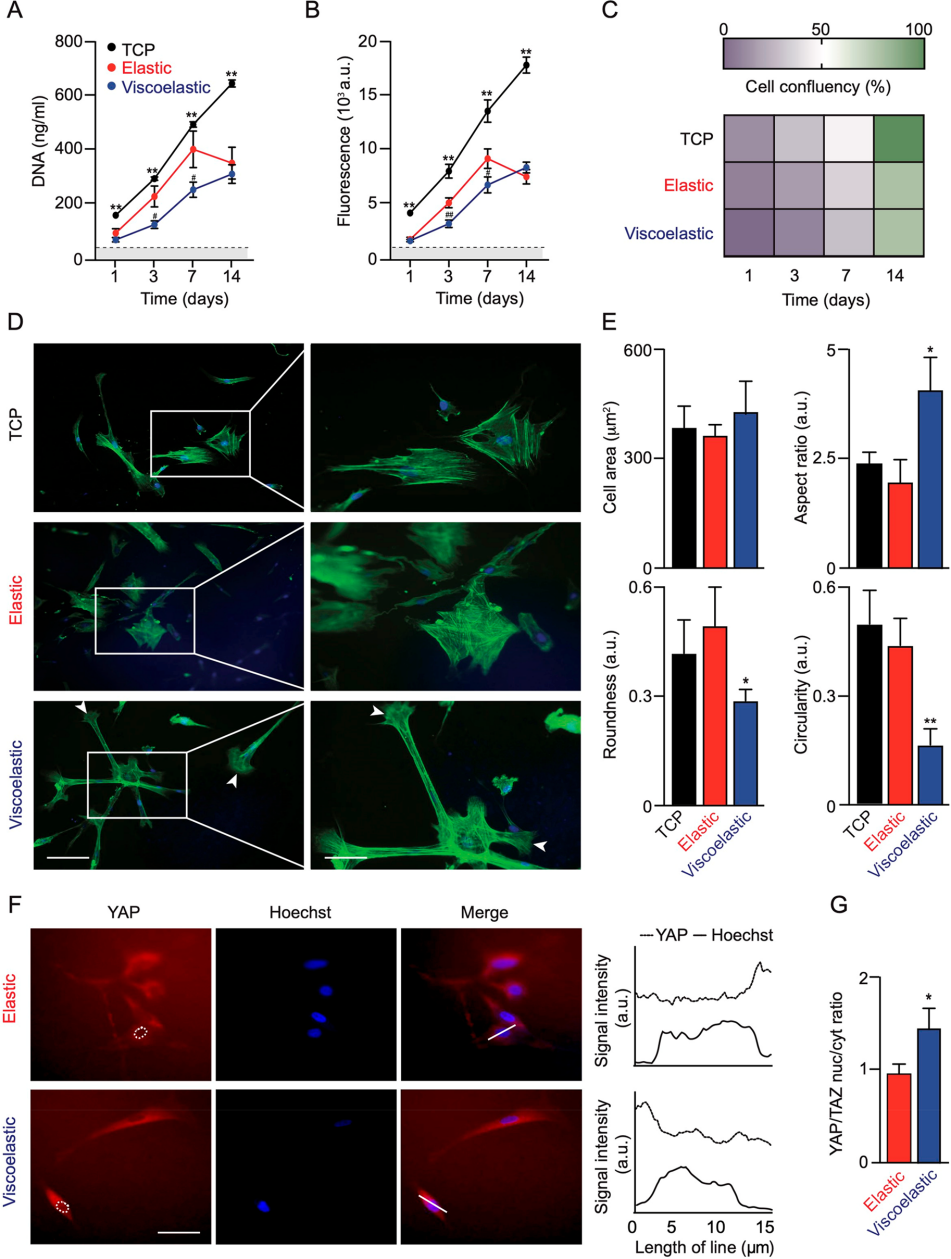
Impact of substrate mechanics on MSC proliferation and morphology. (A) MSC proliferation, (B) metabolic activity, and (C) confluency at day 14. Data were analyzed from four MSC donors and presented as mean ± SD, *n* = 4 independent experiments. Error bars are hidden in the plot symbols when not visible; *p ≤ 0.05 and **p ≤ 0.01 comparison of silk hydrogels with tissue culture plastic (TCP) control. For ^#^*p* ≤ 0.05 and ^##^*p* ≤ 0.01 comparison of elastic and viscoelastic silk hydrogel cultures at the respective time point. (D) Representative images of cytoskeletal F-actin staining and (E) quantification of morphological characteristics of MSCs (92 cells in *n* = 21 images from three pooled experiments). For *p ≤ 0.05 and **p ≤ 0.01 comparison of silk hydrogels with control cultures. Scale bar 20 μm. (F) Representative images of YAP staining and (G) quantification of the nuclear-to-cytoplasmic ratio of YAP (50 MSCs in *n* = 18 images from three pooled experiments). For *p ≤ 0.05 comparison of elastic and viscoelastic silk hydrogel cultures. Scale bar, 20 μm. Dashed white lines represent nuclear outlines. The 15 μm line in the merged images were used for the profile plots to highlight nuclear localized YAP/TAZ.

The MSC response to substrate mechanics was monitored for up to 14 days. The DNA content was used to quantify cell proliferation. During the first 7 days, the MSCs showed a similar proliferation curve to the proliferation seen on plasma-treated tissue culture polystyrene, but the proliferation then stagnated, whereas the control cells continued to proliferate. The MSCs cultured on elastic and viscoelastic silk hydrogels showed different growth profiles, as the MSCs cultured on viscoelastic silk hydrogels had the slowest growth kinetics but eventually caught up with the cells on elastic hydrogels at day 14 (Figure 2A).

Mitochondrial activity was also assessed in the MSCs, and the profiles closely mirrored the DNA content (Figure 2B). Semiquantitative assessment of cell proliferation showed similar confluency across all substrates during the first 3 days (Figure 2C). At day 14, the MSCs on both silk hydrogel substrates showed 80% confluency, whereas control cultures were 100% confluent (Figure 2C). The substrate stress relaxation had similar effects on cell spreading and cell proliferation, and a comparison of these data sets revealed no significant correlations among the tested (data not shown). (At day 3, cell spread versus cell proliferation on elastic hydrogels, *R*^2^ = 0.6850, *p* = 0.3749; cell spread versus cell proliferation on viscoelastic hydrogels, *R*^2^ = 0.1625, *p* = 0.7359).

On viscoelastic hydrogels, the MSCs at day 3 had assumed a more stretched and elongated morphology when compared to MSCs on elastic silk hydrogels. MSCs cultured on viscoelastic hydrogels showed signs of membrane protrusion, with intense local actin polymerization. These features were absent in MSCs cultured on elastic hydrogels or on the tissue culture plastic control substrate (Figure 2D). For both elastic and control substrates, the cells adopted a more cuboidal morphology. Quantification of the cell area, aspect ratio, roundness, and circularity revealed that the MSCs cultured on viscoelastic hydrogels had significantly greater cell areas and aspect ratios and concordantly lower roundness and circularity when compared to cells growing on elastic hydrogels (Figure 2E).

The influence of the ligand density on the cell attachment and cell spreading of hMSCs was established by quantifying fibronectin (FN) adsorption onto the surface of both hydrogel types. The surface density of the adsorbed FN increased with the solution concentration. The FN adsorption was significantly higher on the surface of the viscoelastic hydrogels than on the elastic hydrogels at 6 h for the low FN concentration (10 ng/mL) and at 1 h for the high FN concentration (100 ng/mL) (Figure S4). At 24 h, the FN adsorption on the surfaces of both hydrogel types was comparable, with no statistically significant differences.

The possibility that stress relaxation of the hydrogel substrates could alter downstream behaviors of hMSCs was assessed through evaluation of the Yes-associated protein/transcriptional coactivator (YAP/TAZ) mechanosensitive signaling pathways by determining the nuclear translocation of YAP.^34^ The translocation of YAP from the cytoplasm into the nucleus became more apparent in hMSCs cultured on viscoelastic hydrogels than those on elastic silk hydrogels (0.5-fold increase in the ratio of nuclear to cytoplasmic YAP/TAZ) (Figure 2F,G).

### 3.3 Gene and Protein Expression in Response to Mechanics

**Figure 3:**
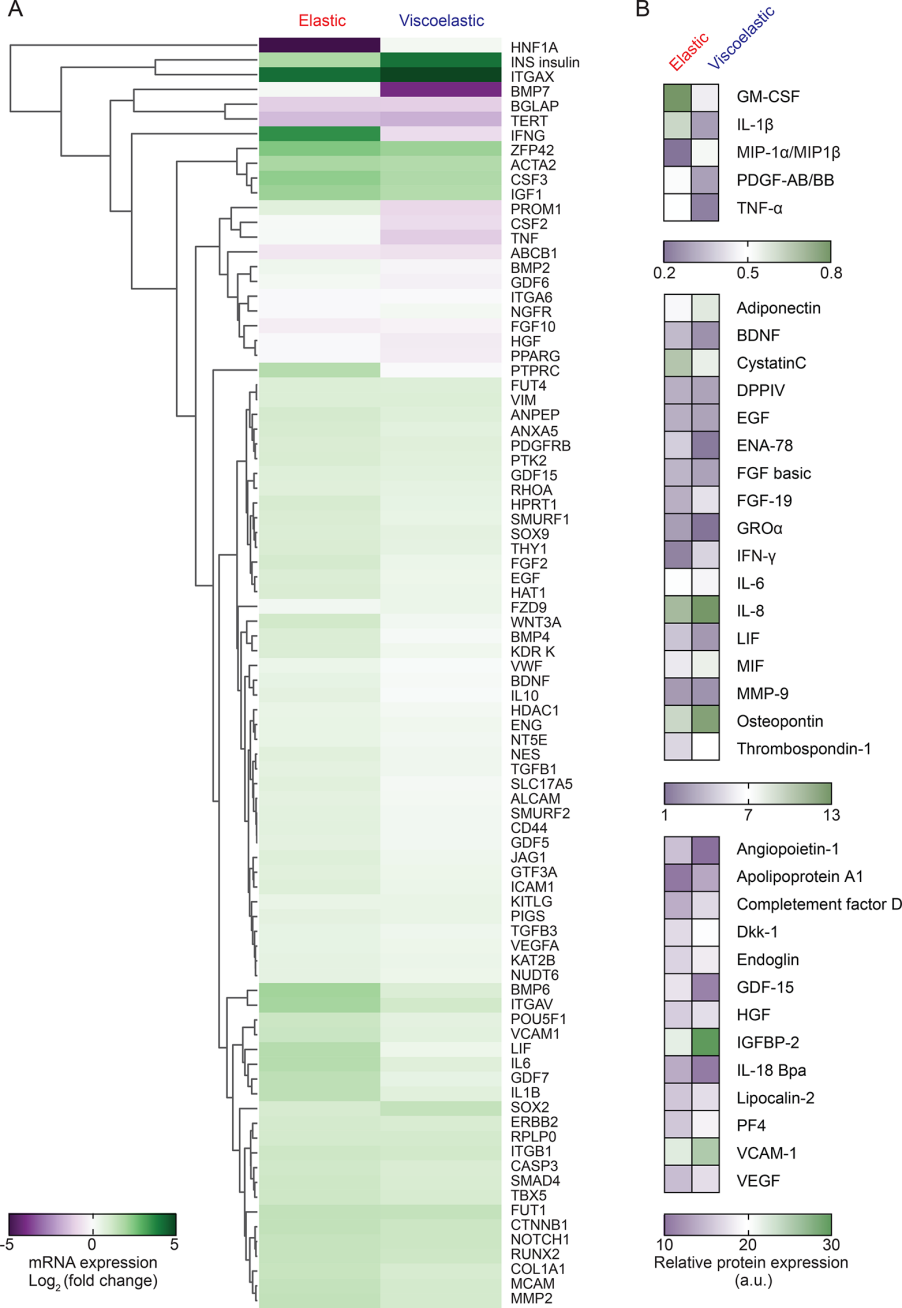
Impact of substrate mechanics on MSCgene and secretome expression. (A) Hierarchical cluster analysis of gene expression of MSCs cultured on silk hydrogels for 14 days using pooled RNA isolation from four MSC donors. Differential gene expression was calculated and shown as log2 fold change. (B) Expression pattern of secreted proteins over 14 days. A conditioned culture medium was pooled from four MSC donors prior to analysis.

The impact of culture substrates on MSC mRNA expression was assessed using pooled MSCs from four donors to minimize donor variability. The MSC response to elastic and viscoelastic hydrogels was characterized following 14 days of substrate priming (Figure 3A). The overall gene expression patterns for MSCs cultured on elastic hydrogels and viscoelastic hydrogels differed substantially, as the patterns for MSCs cultured on elastic hydrogels were clustered more closely to the patterns for the tissue culture controls than for the cells growing on viscoelastic hydrogels. Gene expression of *IL-1β*, *IL-6*, *LIF*, *BMP-6*, *BMP-7*, and *protein tyrosine phosphatase receptor type C* were substantially higher in MSCs cultured on elastic hydrogels than those on on viscoelastic hydrogels, whereas this pattern was reversed for *insulin*, *HNF-1A*, and *SOX-2*. When compared to the tissue culture plastic controls, cells growing on both hydrogels showed an upregulation of *CSF3*, *IGF1*, *integrin alphaV* and *actin alpha2* and downregulation of *bone gamma-carboxyglutamate protein* (*BGLAP*), *telomerase reverse transcriptase* (*TERT*), and *tumor necrosis factor* (*TNF*). Across all three substrate types, the expression patterns of the cytoskeleton-related markers *integrin β1*, *vimentin*, *RhoA*, and *catenin beta1* (CTNNB1) were similar or increased for silk culture substrates (Figure 3A, Figure S6). By contrast, *integrin alphaX* was only expressed by hydrogel-cultured MSC and was absent in the tissue culture plastic control cultures.

The IPA software was used to generate interactive networks in response to the different substrates to better explain the biological relevance of the expressed genes. The overall trends were similar for both elastic and viscoelastic hydrogels. Nonetheless, some notable differences were observed in two pathways, and especially in the *IL-1β* canonical pathway (Figure S5). To obtain further insights, we clustered the differentially expressed genes and predicted the potential functional canonical pathway networks using IPA core analysis. The top five canonical pathways enriched in the differentially expressed genes included cellular development, cell-to-cell signaling and interaction, and cell death and cell survival, and also connective tissue and nervous system development and function (Table S1). Subsequently, the most consistent network-related gene expression in MSCs cultured on elastic hydrogels involved glucose metabolism disorders and inflammatory responses (consistency score +2.83 and +1.789, respectively), thereby predicting the regulation of these functions by an activation of *CRYAB*, *NR1H2*, and *PIM1* and by an inhibition of *IRF1* and *NR1/2*. By contrast, a culture on viscoelastic hydrogels was associated with homing, fatty acid metabolism, and chemotaxis (consistency score +3.50, + 3.32, and +3.21, respectively), mainly mediated through MAPKs and CHUK (Tables S2 and S3).

The expression of selected target genes was also verified individually across the four hMSC donors (Figure S6). These results showed similarities across all the donors, as well as with the data sets generated from gene array analyses. For example, for all the donors the expression of *IL-1β*, *IL-6*, *integrin alphaV*, and *LIF* was significantly higher with elastic hydrogels than those with viscoelastic hydrogels, whereas *VCAM1* expression was significantly greater with viscoelastic hydrogels than that with elastic hydrogels (Figure S6). The cytoskeleton-related genes, such as *integrin β1* and *RhoA*, showed no significant differences between the two hydrogel types. *Integrin alphaV* was upregulated with both hydrogels compared to that with plasma-treated tissue culture plastic. Expression of *MMP2* was unchanged for either hydrogel substrate compared to that for plasma-treated tissue culture plastic.

The expression data sets were complemented with protein secretion profiles of the MSCs. Of the 108 analyzed proteins, 35 showed differential expression profiles in response to stress relaxation (Figure 3B and Figure S7). The proteomic profiles of cells cultured on elastic and viscoelastic hydrogels shared some common proteins that were secreted at high levels from the MSCs (e.g., FGF basic, IL-8, HGF, IGFBP-2, EGF, Endoglin, and VEGF). By contrast, elastic hydrogels induced the production of the cytokines angiopoietin-1, BDNF, LIF, FGF basic, GDF-15, ENA-78, GRO alpha, complement factor D, IL-18 Bpa, and cystatin C. Growth on the viscoelastic hydrogels induced protein expression of apolipoprotein, MIF, thrombospondin, osteopontin, VEGF, IL-8, PDGF AB/BB, IGFBP-2, and VCAM-1. Moderately elevated signals for Dkk-1, DPP IV, lipocalin-2, PF4, adiponectin, and FGF-19 were found in cells growing on viscoelastic hydrogels compared to cells growing on elastic ones. Notably, a strong increase was observed in both gene expression and protein levels for the pro-inflammatory cytokines IL-1β, and LIF in the MSCs cultured on elastic hydrogels.

### 3.4 MSCs Exometabolome Changes in Response to Mechanics

**Figure 4:**
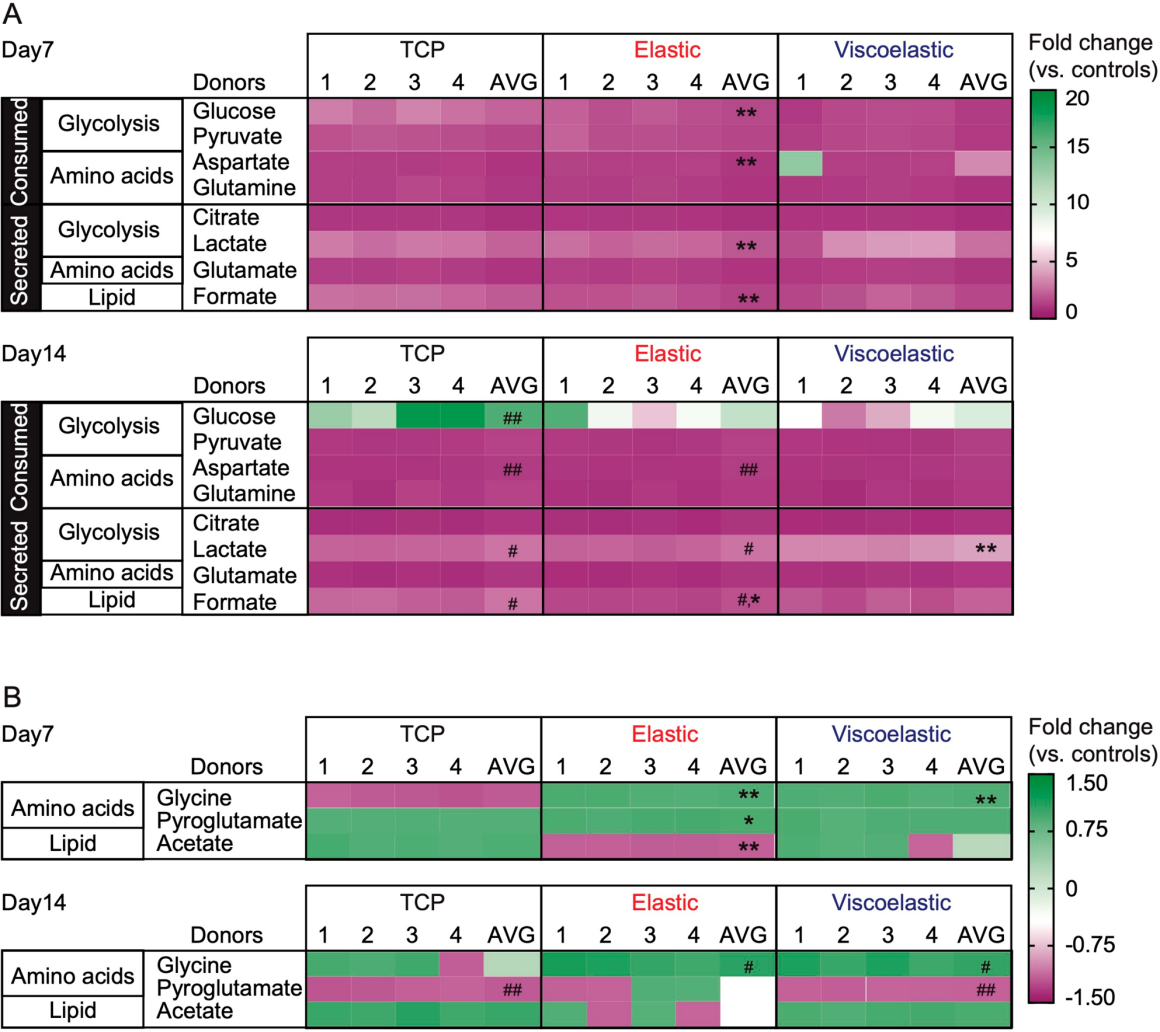
Impact of substrate mechanics on MSC metabolism. Exometabolites of silk hydrogel MSC cultures at 7 and 14 days. The criterion for including a metabolite in the heatmap was (A) absolute fold change ≥1.05 grouped as distinct consumed or secreted metabolites, and (B) absolute fold change ≤ 1.05 represented as negative values for consumptions and positive values for secretions. Color codings are expressed as relative fold changes to matched acellular medium samples (negative values for consumptions; positive values for secretions). Control cultures used tissue culture plastic (TCP) as the culture substrates. For all treatment groups, a conditioned culture medium was collected from four MSC donors (denoted 1–4) and analyzed individually and combined (AVG: average). For statistical analyses ^#^*p* ≤ 0.05 and ^##^*p* ≤ 0.01 impact of time for the respective culture substrate, whereas *p ≤ 0.05 and **p ≤ 0.01 denotes a comparison between the substrates and control at the respective time point.

The metabolic response to the culture substrate was monitored by analyzing a conditioned cell culture medium (Figure 4). The data were shown as fold changes compared to the respective acellular media under the same incubation conditions, at days 7 and 14 for all four MSC donors. Control cells consistently consumed glucose, pyruvate, glutamine, and aspartate, while secreting lactate, formate, and glutamate at day 7, together with citrate and acetate at day 14. Cells growing on silk hydrogels showed most of these variations, although with different magnitudes. At both time points (but especially at day 7), the silk-cultured cells displayed lower metabolic activity than the control cells, as evidenced by the smaller fold changes in metabolite levels compared to those of acellular media. Only aspartate was consumed more appreciably by the silk-cultured cells. The levels of glycine were also higher in the medium of the silk-grown cells than those in the control medium. A comparison of the two hydrogels revealed a higher consumption of glucose and glutamine, together with a higher secretion of lactate, by cells growing on the elastic substrate than those on the viscoelastic substrate.

## 4 Discussion

Model systems have contributed to our understanding of MSCs and culture materials and have provided a blueprint for the material design space. However, translating these findings requires materials that are suitable for use in humans. Silk fibroin is a clinically approved biopolymer, and a first-generation viscoelastic silk hydrogel assembled from regenerated silk fibroin was approved in 2019 for use in humans. This milestone now catalyzes new bench-to-bedside translational opportunities. We have selected silk fibroin with different cross-linking modes to assess how hydrogel stress relaxation can prime MSC behavior in two dimensions. We have explored the MSC biological responses toward elastic and viscoelastic silk hydrogels in studies with human MSCs from four healthy donors to minimize potential donor variability.

The solid content is known to directly influence the mechanical properties of silk hydrogels.^21,25,29^ Hydrogels with a greater mechanical strength are obtained at a higher silk content. To facilitate handling and a robust solution–gel transition, we used 4% (w/v) silk fibroin. The resulting secondary structure of our silk hydrogels was similar to previously reported structures.^25,29^ For elastic hydrogels, the cross-linker concentration was fine-tuned to yield an initial elastic modulus of 1 kPa (Figure S3B) (Table S4), whereas matched viscoelastic hydrogels were assembled using physical cross-linking. The selected initial elastic modulus of 1 kPa is physiologically relevant^5^ and is implicated in MSC self-renewal and reduced replicative senescence.^35^ Recent work with nondegradable alginate hydrogels showed decoupling of irreversible creep from stress relaxation and modulus, thereby demonstrating that network plasticity drove cell spreading.^36^ Cell spreading impacts cell behavior, whereas mechanical remodeling of the extracellular matrix, including matrix degradation, often occurs in health and disease, ultimately impacting cell biology. Although the silk biopolymers of the hydrogels are biodegradable, no signs of substrate degradation were evident during cell culture in our study. Importantly, the expression of the silk proteolytic enzyme MMP2 was unchanged for either hydrogel substrate compared to that of plasma-treated tissue culture plastic, suggesting that silk hydrogel mechanics, rather than hydrogel degradation, were responsible for the observed biological differences.

Both silk hydrogel types supported MSC proliferation, although elastic hydrogels supported greater cell proliferation (but still outpaced by polystyrene controls). Previous studies have shown that elastic silk hydrogels with a comparable stiffness and silk content could support human MSC attachment and proliferation and were able to compete with tissue culture plastic controls.^29^ The underlying reasons for these subtle but distinct differences between our work and previous reports is not clear. However, cell heterogeneity and culture conditions can impact performance. For example, human corneal epithelial cells cultured on chemical versus physically cross-linked silk hydrogels showed better growth on chemically cross-linked hydrogels, although the presence of serum in the culture medium abolished these effects.^37^ By contrast, stress relaxation of alginate hydrogels increased mouse myoblast proliferation when compared to that of elastic controls.^38^ MSC adhesion to silk is likely mediated both by factors present in the serum (e.g., fibronectin) and by factors secreted from the MSCs themselves (i.e., collagen type I) that adsorb to the silk substrate. We observed similar amounts of fibronectin adsorption to elastic and viscoelastic silk hydrogels, suggesting that the secondary structure and microstructure of silk hydrogels had no significant effect on the protein–surface adsorption.

Increasing the stiffness of purely elastic substrates contributes to cell spreading, cytoskeleton organization, and focal adhesion.^5^ Stiffer substrates promote cell spreading by maintaining tensional homeostasis. For example, soft silk hydrogels (16 kPa) promoted less cell spreading than did stiffer ones (64 kPa).^21^ However, we observed striking differences in morphology for cells grown on viscoelastic versus elastic silk hydrogels, independent of the elastic modulus. Previous work using alginate hydrogels proposed that hydrogels with fast stress relaxation reduced mechanical confinement and enhanced ligand clustering, leading to greater cell spreading.^39^ Similar observations have been reported for other model substrates. For example, MSC spreading increased with a greater loss modulus by increasing Rac1 and N-cadherin expression that, in turn, increased motility and lamellipodial protrusion.^40,41^ We observed no difference in vimentin expression, so we therefore speculate that stiffness, rather than viscoelasticity, is important for regulating vimentin expression. This speculation is supported by the observation that MSCs cultured on stiff gelatin hydrogels showed increased vimentin and decreased tropomyosin cytoskeleton protein expression when compared to soft hydrogels.^42^

Growth of MSCs on elastic hydrogels induced *IL-1β* signaling with the highest IPA network score. We observed increased *IL-1β* gene and protein expression in MSCs grown on elastic silk hydrogels. Previous work has linked *IL-1β* signaling to cell volume regulation via adhesion-independent mechano-transduction, which ultimately impacts differentiation.^43^ Viscoelastic alginate hydrogels supported chondrocyte development, whereas elastic ones restricted chondrocyte volume expansion via *IL-1β* signaling, which negatively regulated chondrocyte gene expression and cell survival.^43^ Consequently, designing therapeutic silk biomaterials to either encourage (or suppress) *IL-1β* signaling could represent a new tissue engineering approach. For example, increases in *IL-1β* signaling could promote normal wound repair during the inflammatory phase of healing.^44^

*IL-1β* signaling also impacts other downstream signaling pathways (Figure S5). For example, integrin β1 receptors are responsible for cell-ECM binding through RGD ligand clustering that, in turn, activates YAP/TAZ, which is implicated in mechanobiology.^7,39,45^ Cells sense substrate mechanics through actomyosin contractility through a mechanism often mediated through Rho^46^ and Rac signaling^41^ that can impact linage commitment. For example, low levels of activated RhoA commit hMSCs to become adipocytes, whereas constitutive expression of activated RhoA protein promotes osteogenesis.^47^ Our study demonstrated an increased expression of integrin β1 and RhoA in MSCs cultured on both viscoelastic and elastic substrates compared to the controls. IPA downstream effector analysis revealed a direct involvement of PPAR gamma transcription factor with RhoA activation in MSCs grown on an elastic substrate but not those on a viscoelastic one. Additionally, monitoring the nuclear localization of mechanically sensitive transcription regulators (e.g., YAP/TAZ) provided compelling evidence that fast-relaxing substrates enhanced downstream mechanosensitive signaling via the RhoA and/or YAP/TAZ signaling pathways, ultimately priming the MSCs.^34^

Substrate mechanics impacts both MSC gene and secretome expression^48,49^ that are implicated in tissue repair. For example, MSCs cultured on stiff polyethylene glycol/hyaluronic acid/gelatin hydrogels showed upregulated VEGF, urokinase plasminogen activator, and IL-8 when compared to cells grown on soft hydrogels.^31^ Physically cross-linked silk hydrogels with an elastic modulus of 10 kPa promoted brain injury repair via TGF β1 secretions,^23,26^ and TGF β1 production has also been reported as important in bone tissue engineering.^50^ We also observed a substantial *TGF β1* increase in MSCs growing on both elastic and viscoelastic silk hydrogels, whereas *BMP-7* was differentially expressed in response to elastic versus viscoelastic silks. Therefore, mechanically tuned silk hydrogels are expected to further enhance bone regeneration beyond the current state of the art.^19^

We also observed other differentially expressed transcriptome and secretome profiles in response to substrate mechanics. For example, apolipoprotein A1, insulin-like growth factor binding protein 2 (IGFBP2), and VCAM 1 were strongly expressed in MSCs growing on viscoelastic silk hydrogels, whereas growth/differentiation factor 15 (GDF15) was higher in MSCs growing on elastic silk hydrogels. The GDF15-stress response cytokine, which belongs to the TGF β1 superfamily, is strongly upregulated during tissue injury.^51^ In agreement with the IPA core analyses, gene expression in MSCs cultured on elastic silk hydrogels was primarily involved in regulatory effects on inflammation, whereas growth on viscoelastic silk hydrogels affected the chemotaxis and fatty acid metabolism networks (Tables S2 and S3).

Assessment of metabolic pathways is crucial for obtaining a better understanding of the cellular responses to substrate mechanics, especially in cells proposed for regenerative therapies.^52^ Growth on silk hydrogels significantly changed the magnitude of consumption of glucose, pyruvate, and some amino acids (mainly glutamine and aspartate), along with the amounts of secreted metabolites (lactate, glutamate, formate, citrate, and acetate). MSCs exposed to silk hydrogels consumed less glucose and secreted less lactate compared to those of controls, suggesting a lower glycolytic flux. Similar reductions were observed for glutamine consumption and glutamate excretion, suggesting decreased glutaminolytic activity. However, consumption of pyruvate, the main fuel for the TCA cycle, was only substantially decreased in cells growing on viscoelastic hydrogels. Interestingly, aspartate consumption from the medium was higher in MSCs growing on silk hydrogels than in control cells. We speculate that this increased aspartate uptake replenished the TCA cycle via the aspartate arginosuccinate shunt used by cells like inflammatory macrophages.^53^ Cells exposed to silk hydrogels for 14 days also showed a decreased secretion of citrate (a TCA cycle intermediate) and of acetate, a metabolite that may be produced from glycolytic pyruvate, especially under conditions of metabolic overflow.^54^ These lower releases of citrate and acetate also hint at possible effects on lipid metabolism, a hypothesis to be verified in future studies. Finally, glycine levels increased only in the medium of cells exposed to silk hydrogels (14 days) and not in silk-containing acellular media, indicating that this variation was not attributable to passive glycine leakage. Instead, glycine excretion must have been triggered via cellular events (e.g., metabolism). This result is similar to observations made previously in macrophages exposed to silk nanoparticles.^55^

## 5 Conclusions

We have examined the impact of silk hydrogel stress relaxation on human MSC biology. Both elastic and viscoelastic silk hydrogels supported cell proliferation but impacted several aspects of cell biology, including morphology, metabolism, and gene and protein expression. Data sets subjected to pathway analysis highlighted that silk hydrogel mechanics primed MSC biology. For example, elastic cultures activated IL-1β signaling in response to hydrogel mechanics. An elastic substrate also induced higher consumption of glucose and glutamine, coupled with a higher secretion of lactate, than that observed in MSCs grown on viscoelastic substrate. However, both silk hydrogels significantly changed the magnitude of consumption of glucose, pyruvate, glutamine, and aspartate, and also metabolite secretion, resulting in an overall lower metabolic activity than that found in control cells. Overall, this study demonstrated that silk hydrogel mechanics impacts MSC biology in two dimensions. Therefore, the fine-tuning of silk hydrogels has the potential to maximize MSC performance.

## Supporting Information Available

Schematic representation of morphological cell metrics; structural analyses of elastic and viscoelastic silk hydrogels; rheological properties; protein adsorption to silk hydrogels; pathway analysis of MSC exposed to elastic and viscoelastic silk hydrogels; qRT-PCR differential gene expression; impact of stress relaxation on MSCsecretome; tables of regulated effector networks (PDF)

## Notes

All data supporting this research are openly available from the University of Strathclyde-Pure at https://doi.org/10.15129/cc18971e-9c85-406e-9b4c-e39b13afd132.
