# Supplementary material for: Silk Hydrogel Substrate Stress Relaxation Primes Mesenchymal Stem Cell Behavior in 2D
Source: ACS Appl Mater Interfaces. 2021 Jun 25;13(26):30420–33. doi: 10.1021/acsami.1c09071 (PMC8289244; doi:10.1021/acsami.1c09071)
Supplement: Supplementary file 1 — am1c09071_si_001.pdf [file am1c09071_si_001.pdf]

Silk Hydrogel Substrate Stress Relaxation Primes Mesenchymal Stem Cell Behavior in 2D

Suttinee Phuagkhaopong, Luís Mendes, Katrin Müller, Manja Wobus, Martin Bornhäuser, Hilary V. O. Carswell, Iola F. Duarte, and F. Philipp Seib*

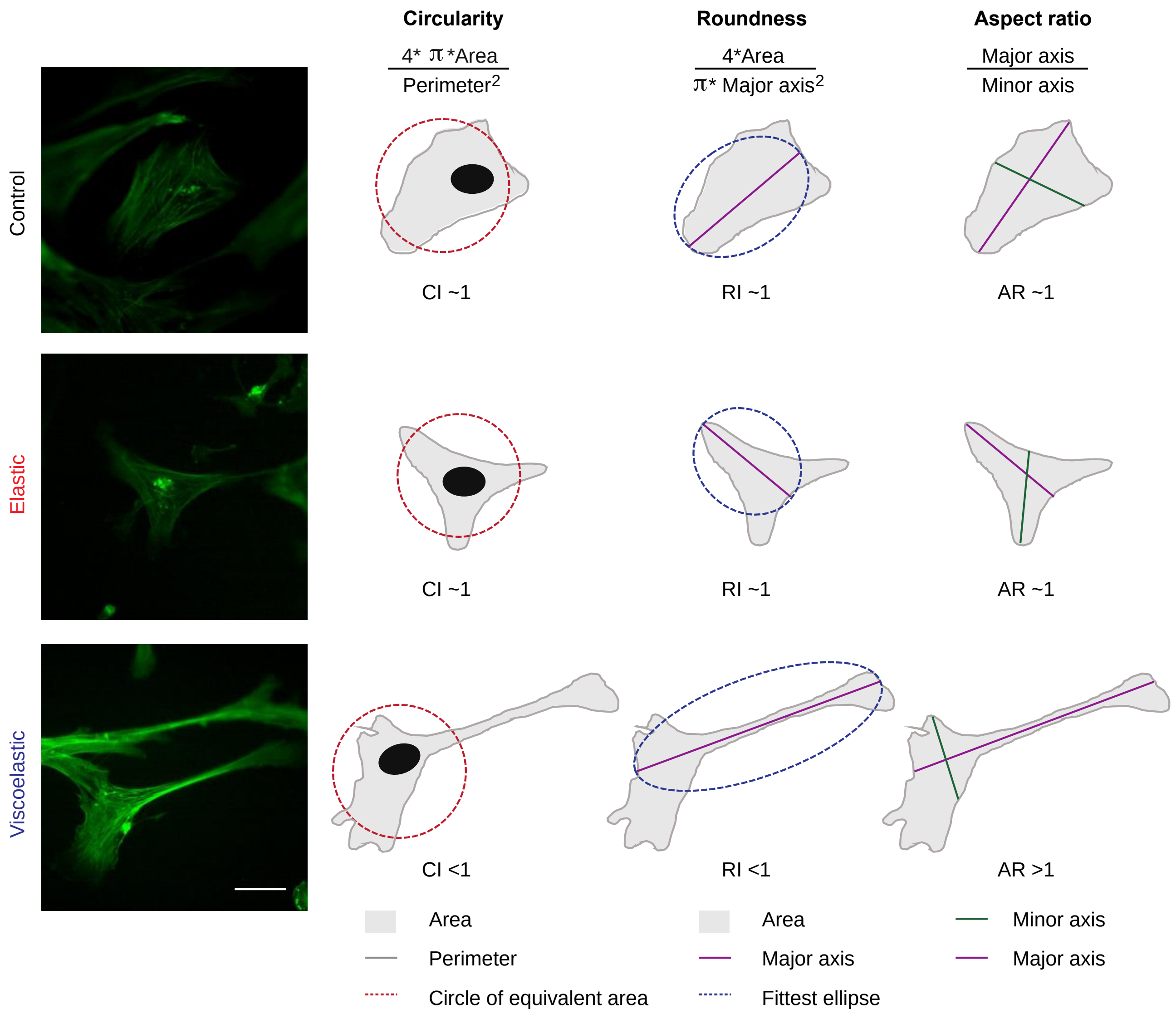

Figure S1. Schematic representation of morphological metrics used to quantify cell area, circularity, roundness, and aspect ratio, using ImageJ software. CI, circularity; RI, roundness; AR, aspect ratio. Scale bar = 10 μm. Figure adapted from reference[32]

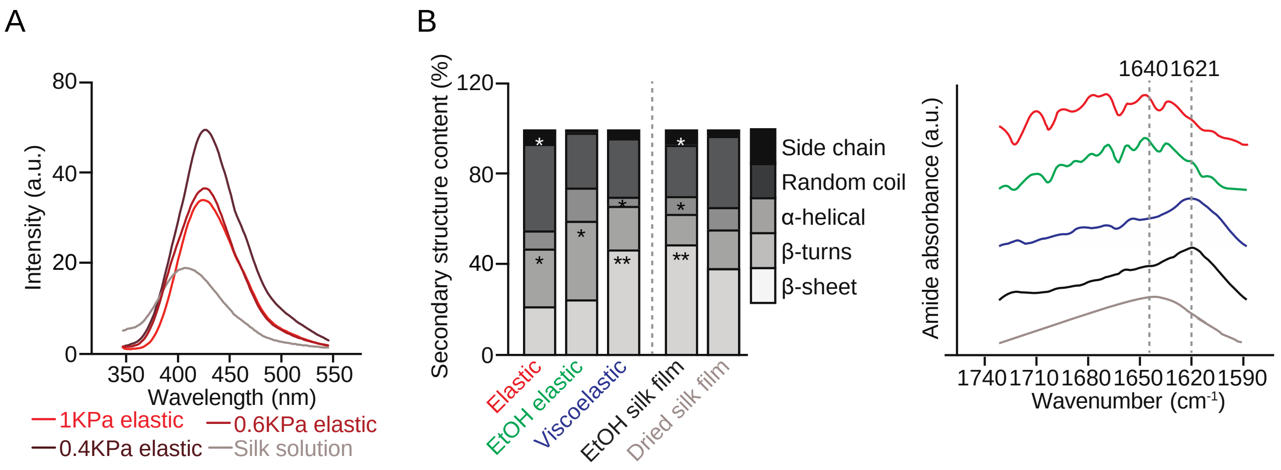

Figure S2. Structural analyses of elastic and viscoelastic silk hydrogels. (A) Monitoring the dityrosine bond peak at 425 nm of elastic silk hydrogels of various stiffnesses (0.4-1 KPa). (B) Secondary structure contents of silk hydrogels. The amide I region was converted from FTIR spectra. Lines indicate the β-sheet peak (1621 cm^{-1}) and α-helix peak (1640 cm^{-1}). Statistical significances were assigned as $^{*}(p \leq 0.05)$ and $^{**}(p \leq 0.01)$ for secondary structure analyses between hydrogel samples and dried silk film (i.e. low β-sheet content). The ethanol treated silk film served as a positive control (i.e. high β-sheet content). n=3 independent experiments.

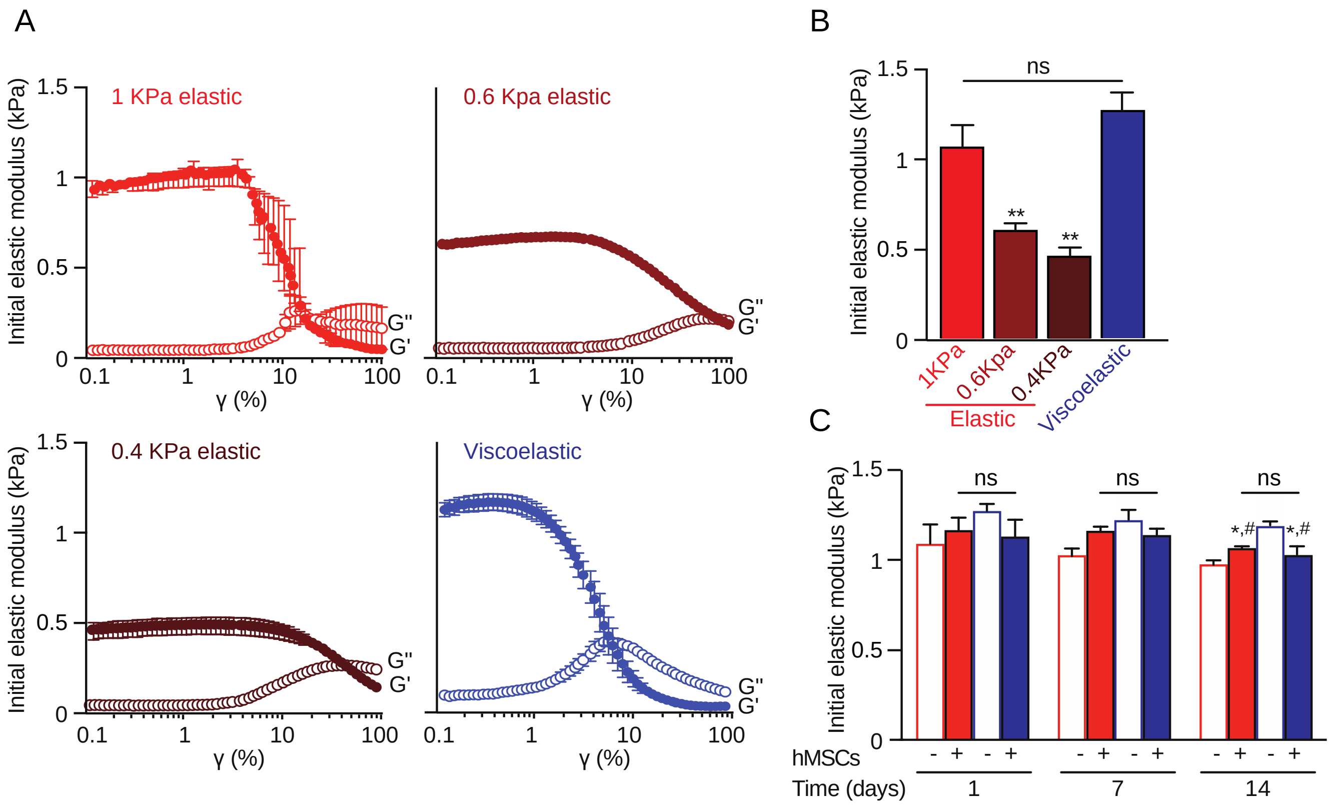

Figure S3. Rheological properties of elastic and viscoelastic silk hydrogels. (A) Strain sweep, storage modulus (G') and loss modulus (G'') curves. Error bars are hidden in the plot symbols when not visible. (B) Initial elastic modulus of elastic and viscoelastic silk hydrogels. Elastic silk hydrogel samples with an elastic modulus of 1.0 kPa, 0.6 kPa and 0.4 kPa were generated using 2.25, 4.5 and 6.75 U/mg horseradish peroxidase, respectively (see the formulations in Table S4). Data are presented as mean ± SD, n=4 independent experiments. **($p \leq 0.01$) comparison among 3 different formulations of elastic silk hydrogels. (C) Initial elastic modulus (~1 kPa) of the hydrogels in the presence and absence of hMSCs for 14 days. Data are presented as mean ± SD, n=5 independent experiments. "ns" indicates no significance. For *($p \leq 0.05$) comparison of silk hydrogels at the same condition either day 7 or day 14 with day 1; # ($p \leq 0.05$) comparison of silk hydrogel with and without cell culture at the respective time point.

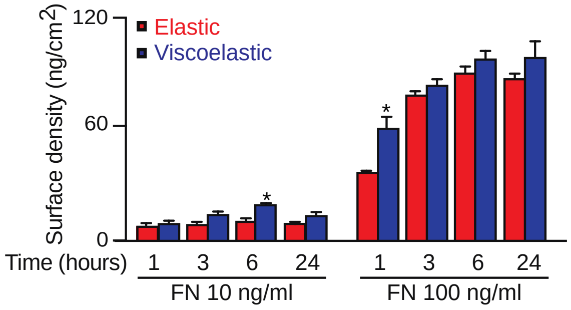

Figure S4. Protein adsorption on silk hydrogels. Surface density of fibronectin (FN) on the hydrogels exposed to 10 and 100 ng/ml of FN. For *($p \leq 0.05$) comparison of elastic and viscoelastic silk hydrogels at the respective FN concentration and time point.

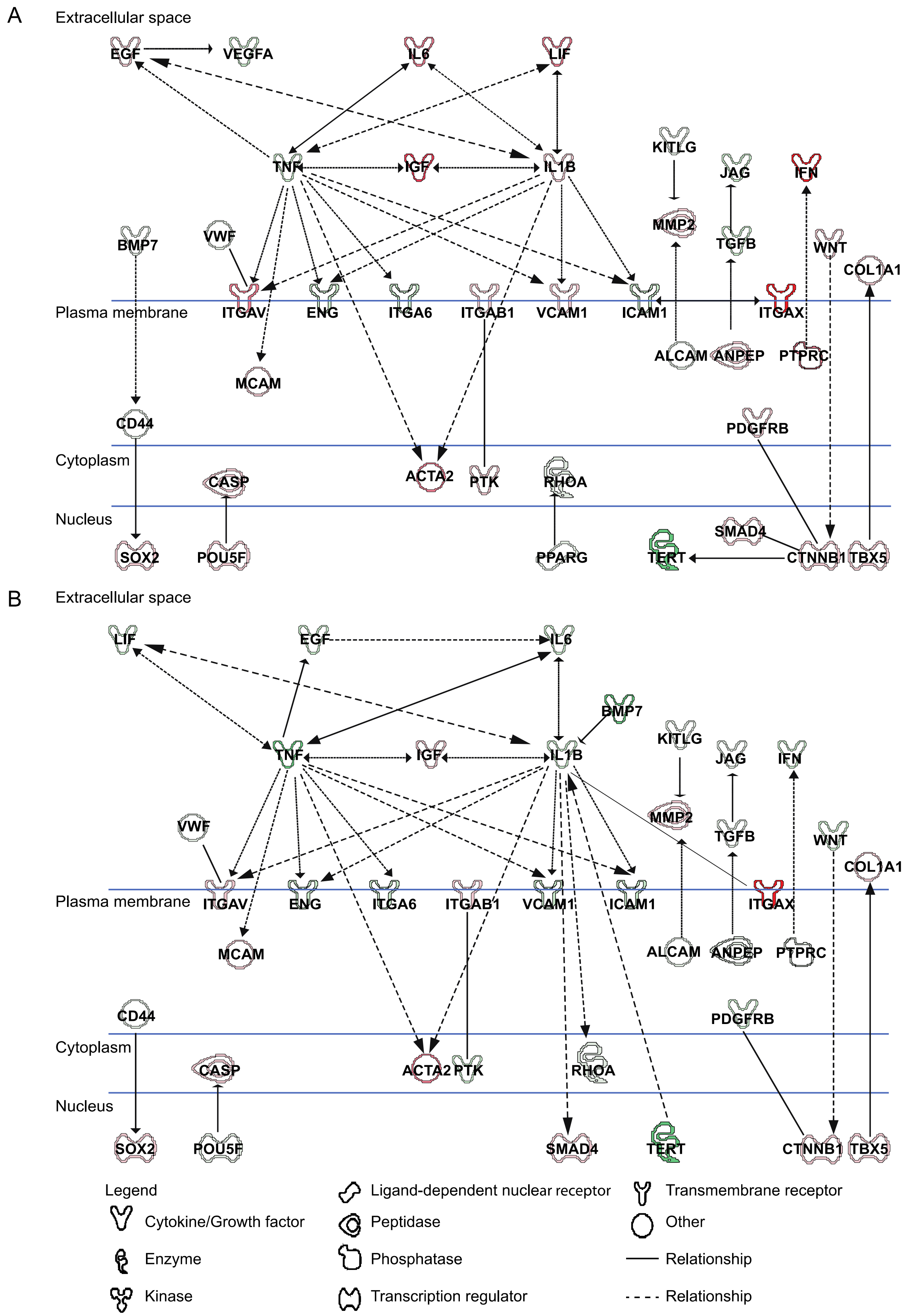

Figure S5. Pathway analysis of MSC exposed to elastic and viscoelastic silk hydrogels. Tissue culture treated polystyrene culture controls were mapped against (A) elastic silk hydrogels and (B) viscoelastic silk hydrogels. The green color denotes a decrease in expression in cell culture on hydrogels relative to control and the red color denotes the respective increase.

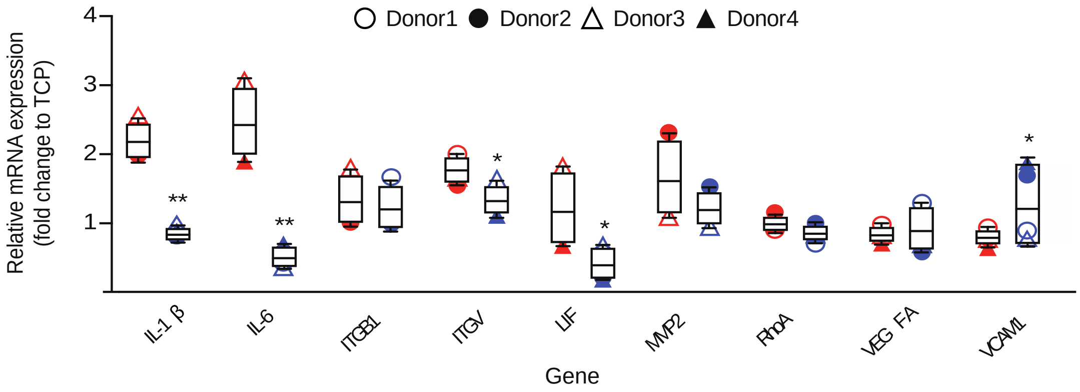

Figure S6. Differential gene expression of hMSCs cultured on mechanically tuned silk hydrogels. Gene expression patterns were determined using RNA extracted from 4 individual healthy donor hMSCs and visualized by a Tukey boxplot. For *($p \leq 0.05$) and **($p \leq 0.01$) comparison of elastic (red) and viscoelastic (blue) silk hydrogels.

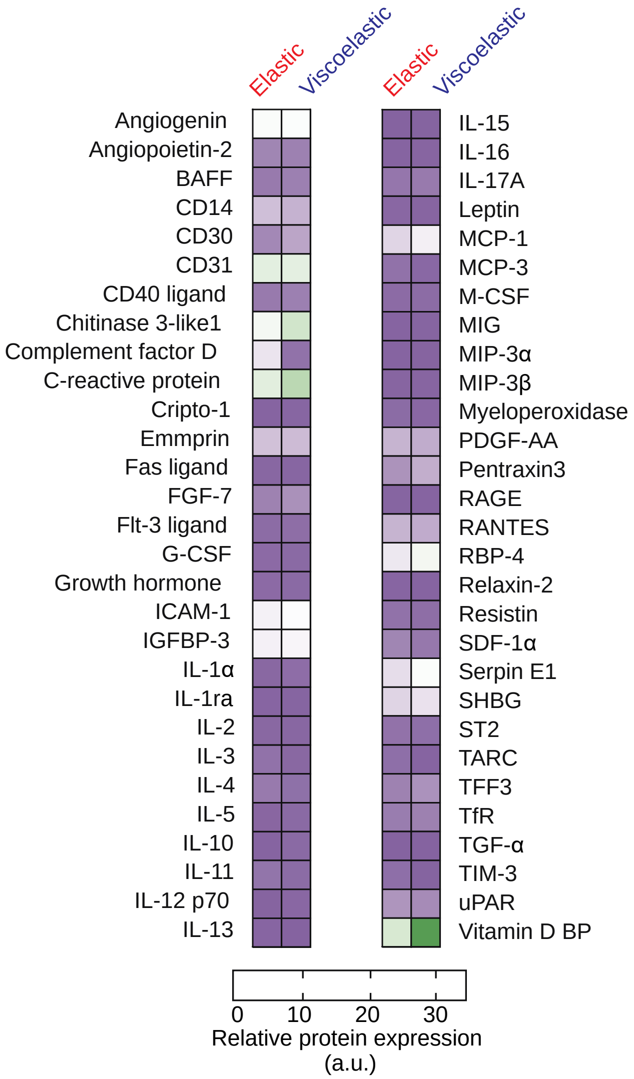

Figure S7. Impact of stress relaxation on MSC secretome. Secreted proteins with similar expression patterns. MSCs were grown on elastic and viscoelastic silk hydrogels for 14 days. The complementary data sets are in Figure 3. Conditioned culture medium was pooled from 4 MSC donors.

Table S1 Top regulator effect networks responsible for cell cultured on elastic and viscoelastic silk hydrogels

Rank	Main molecules in network	Score	Focus molecule	Top functional networks
1	BGLAP, BMP2, BMP4, BMP6, BMP7, ENG, FUT4, GDF15, GDF7, Integrin alpha/beta1, RUNX2, SMAD4, Smooth muscle actin, SOX9, TGFBR	22	12	Cellular development, embryonic development, organismal development
2	CD44, CSF2, CSF3, FGF10, HGF, ICAM1, Interferon alpha, ITGB1, NOTCH1, PTK2, PTPRC, RPLP0, TNF, VCAM1	22	12	Cell-to-cell signaling and interaction, hematological system development and function, inflammatory response
3	CASP3, CTNNB1, ERBB2, FZD9, HDAC1, HNF1A, Insulin, POU5F1, RHOA, SMURF1, SOX2	22	12	Cancer, cellular development, organismal injury and abnormalities
4	ACTA2, ADCY, ADRB, COL1A1, EGF, FGF2, GDF5, GTF3A, IGF1, MMP2, NT5E, NUDT6, PTPase, SMURF2, VEGF	20	11	Connective tissue development and function, organismal injury and abnormalities, tissue morphology
5	ANXA5, BDNF, HPRT1, IL1B, LIF, NGFR, PDGFR, PDGFRB, VEGFA, WNT3A, ZFP42	18	10	Cardiovascular disease, cell death and survival, nervous system development and function

Table S2 Top regulator effect networks responsible for cell cultured on elastic silk hydrogel

Rank	Regulators	Disease & functions	Consistency score
1	CRYAB, NR1H2	Failure of heart,<br>glucose metabolism disorder	2.828
2	Esrra, MMP9	Failure of heart	2.449
3	PIM1	Inflammatory response	1.789
4	IRF1	Glucose metabolism disorder	-3.536
5	NR 1/2	Inflammatory response	-4.158

Table S3 Top regulator effect networks responsible for cell cultured on viscoelastic silk hydrogels

Rank	Regulators	Disease & functions	Consistency score
1	p38 MAPK	Homing cells	3.5
2	ERK1/2	Fatty acid metabolism	3.317
3	ERK1/2	Chemotaxis	3.207
4	CHUK	Chemotaxis	3.175
5	poly rl:rC-RNA	Attachment of cells	3.015

Table S4 Various HRP concentrations used for elastic silk hydrogels in total volume of 1 ml of 4% (w/v) silk solution

Conditions	HRP (μl)	H_2O_2 (μl)	Unit of HRP/mg of silk fibroin
0.4KPa elastic	450	450	6.75
0.6KPa elastic	300	300	4.5
1KPa elastic	150	150	2.25
